# Editorial: Rising stars in translational endocrinology

**DOI:** 10.3389/fendo.2023.1266825

**Published:** 2023-08-11

**Authors:** Leandro Kasuki

**Affiliations:** ^1^ Neuroendocrinology Research Center/Endocrinology Division, Hospital Universitário Clementino Fraga Filho, Universidade Federal do Rio de Janeiro, Rio de Janeiro, Brazil; ^2^ Neuroendocrinology Division, Instituto Estadual do Cérebro Paulo Niemeyer, Rio de Janeiro, Brazil; ^3^ Endocrinology Division, Hospital Federal de Bonsucesso, Rio de Janeiro, Brazil

**Keywords:** translational medicine, DHEA, polycystic ovary syndrome (PCOS), SST2, pericardium tuberculosis

Translational medicine aims to bring the latest discoveries in medical science from bench to bedside (1). Thus, it is of utmost importance as it translates all the advances of basic science in benefit of clinical practice and the same is valid in the endocrinology field. Recognizing the future of translational endocrinology and the researchers that will lead this field in the future is fundamental. In the present Research Topic some of the researchers published their latest discoveries.

Bongiovanni et al. reviewed the actions of dehydroepiandrosterone (DHEA) in infectious disease, especially in tuberculosis. They analyzed *in vitro* studies, mouse models and, finally, data from patients with tuberculosis that show that DHEA is likely beneficial during the infection. They discuss the results of their *in vitro* studies that showed that DHEA seems to be related to mycobacterial elimination in tuberculosis-infected macrophages (2) and also reviewed the favorable effects of DHEA treatment in many diseases, therefore suggesting a potential benefit in the treatment of tuberculosis by reducing drug resistance and the length of modern short-course chemotherapy.

Gurule et al. reviewed the effects of prenatal androgens in the sheep models of polycystic ovarian syndrome (PCOS) on the developmental programming of hypothalamic-pituitary alterations. In these models there are alterations in all three steroid feedback mechanisms controlling GnRH/LH secretion and a marked increase of pituitary sensitivity to GnRH. The knowledge of these alterations may allow the development of new therapeutic targets for the treatment of neuroendocrine disfunction in women with PCOS.

Shenje et al. described for the first time, that elevated rates of cortisol/cortisone are observed in the pericardium of patients with a diagnosis of tuberculosis pericarditis (TBP) in comparison with that observed in the plasma or in the saliva. Interestingly, elevated cortisol/cortisone ratio was associated with a different profile of cytokine response (elevated levels of interferon gamma, tumor necrosis factor-alpha, interleukin-6 and 8 and induced protein 10). The patients were also randomized to receive 120 mg of prednisolone or placebo, and a single dose of prednisolone was sufficient to evoke an immunomodulatory effect. The study raises important questions and future prospective clinical trials are needed to prove if glucocorticoid administration is indeed of benefit in patients with TBP.

Klomp et al. analyzed the expression of somatostatin receptor type 2 (SST2) and the presence of epigenetic markers surrounding the SST2 promoter region in samples of small intestinal neuroendocrine tumors (SI-NETs) and compared with normal small intestinal samples. They observed that SI-NETs have lower SST2 promoter methylation levels and lower H3K27me3 methylation levels compared to normal SI-tissue and also showed a significant negative correlation between SST2 mRNA expression level and the mean level of DNA methylation within the SST2 promoter region in both normal tissue and SI-NET tissue. The expression of SST2 in SI-NETs is of importance for prognosis and treatment of these tumors and, although additional studies are required in this filed, the results help to clarify the regulation of SST2 expression in these tumors.

These Research Topic of four articles in this editorial showcase demonstrate the importance and applicability of translational research in different areas of endocrinology.

## Author contributions

LK: Writing – original draft, Writing – review & editing.
